# Time scale and dimension analysis of a budding yeast cell cycle model

**DOI:** 10.1186/1471-2105-7-494

**Published:** 2006-11-09

**Authors:** Anna Lovrics, Attila Csikász-Nagy, István Gy Zsély, Judit Zádor, Tamás Turányi, Béla Novák

**Affiliations:** 1 Laboratory for Chemical Kinetics, Institute of Chemistry, Eötvös University (ELTE), Pázmány P. sétány 1/A, H-1117 Budapest, Hungary; 2 Molecular Network Dynamics Research Group of the Hungarian Academy of Sciences and Budapest University of Technology and Economics, Gellért tér 4, H-1521 Budapest, Hungary

## Abstract

**Background:**

The progress through the eukaryotic cell division cycle is driven by an underlying molecular regulatory network. Cell cycle progression can be considered as a series of irreversible transitions from one steady state to another in the correct order. Although this view has been put forward some time ago, it has not been quantitatively proven yet. Bifurcation analysis of a model for the budding yeast cell cycle has identified only two different steady states (one for G1 and one for mitosis) using cell mass as a bifurcation parameter. By analyzing the same model, using different methods of dynamical systems theory, we provide evidence for transitions among several different steady states during the budding yeast cell cycle.

**Results:**

By calculating the eigenvalues of the Jacobian of kinetic differential equations we have determined the stability of the cell cycle trajectories of the Chen model. Based on the sign of the real part of the eigenvalues, the cell cycle can be divided into excitation and relaxation periods. During an excitation period, the cell cycle control system leaves a formerly stable steady state and, accordingly, excitation periods can be associated with irreversible cell cycle transitions like START, entry into mitosis and exit from mitosis. During relaxation periods, the control system asymptotically approaches the new steady state. We also show that the dynamical dimension of the Chen's model fluctuates by increasing during excitation periods followed by decrease during relaxation periods. In each relaxation period the dynamical dimension of the model drops to one, indicating a period where kinetic processes are in steady state and all concentration changes are driven by the increase of cytoplasmic growth.

**Conclusion:**

We apply two numerical methods, which have not been used to analyze biological control systems. These methods are more sensitive than the bifurcation analysis used before because they identify those transitions between steady states that are not controlled by a bifurcation parameter (e.g. cell mass). Therefore by applying these tools for a cell cycle control model, we provide a deeper understanding of the dynamical transitions in the underlying molecular network.

## Background

**Figure 1:**
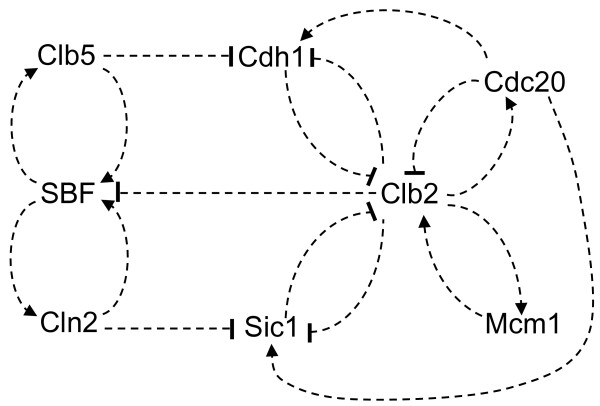
**Molecular interaction map of the budding yeast cell cycle**. The network corresponds to the Chen paper [12]. Lines with arrowheads represent activations, ones with -| represent inhibitory effect. See text for details.

The cell cycle is the sequence of events by which a growing cell replicates all of its components and divides them into two daughter cells [1]. Proliferating cells are repeating this sequence therefore the process is periodic. The eukaryotic cell division cycle is driven by an underlying molecular network which centers around complexes of cyclin-dependent kinases (Cdk's) and cyclins [2,3]. In proliferating cells the cell cycle engine is in periodic motion which suggested to many theoreticians that it is driven by a limit cycle oscillator [4-6]. In our view the cell cycle engine can show limit cycle behavior but only under special developmental contexts like early development [7,8]. In contrast, the cell cycle of growing cells is controlled by checkpoint mechanisms that generate stable steady states [9,10]. As a consequence, the cell cycle progression of growing cells can be viewed as irreversible transitions among stable states [10,11]. The driving force for these transitions is provided by the growth of cytoplasm and at the end of the cycle the cell divides and the control system settles in a steady state where it was starting from. In this paper we try to illustrate this point by using one of the models for the budding yeast cell cycle [12]. The "Chen model" [12] is defined by a 13-variable set of ordinary differential equations (and related algebraic equations) and by 73 kinetic parameters. The kinetic equations describe the dynamics of the core cell cycle regulatory components: different Cdk/cyclin complexes that drive bud formation, DNA replication and mitosis [2,3]; the regulators of cyclin degradation (Cdc20 and Cdh1/Hct1) and synthesis (SBF and Mcm1) and a Cdk inhibitor (Sic1). There are several positive and negative feedback loops among cell cycle control components in the model (Fig. 1). Both Cln2 and Clb2 cyclin synthesis are characterized by transcriptional positive feedback loops because the corresponding Cdk/cyclin complexes (Cln2/Cdc28 and Clb2/Cdc28) activate their own transcription factor (SBF and Mcm1) [13-15]. Another positive (or double-negative) feedback is between Clb2/Cdc28 kinase and its G1 enemies (Sic1 and Cdh1): they inactivate or promote the degradation of each others [16-18]. All the positive feedbacks in the mechanism are counteracted by negative feedback loops (Fig. 1). Cdc28/Cln2 besides activating its transcription factor (SBF) which is a positive feedback, initiates a sequence of events that inhibits SBF: Cln2 -| (Sic1, Cdh1) -| Clb2 -| SBF which is a time delayed negative feedback loop. Similarly, Clb2 kinase which is activated by a transcriptional positive feedback [13], activates Cdc20 that promotes Clb2 degradation (negative feedback). The double-negative feedback is also regulated by a negative feedback, because Clb2 activates Sic1 and Cdh1 via Cdc20: Clb2 → Cdc20 → (Sic1, Cdh1) -| Clb2.

A series of mathematical and computational methods have been developed for the analysis of complex reaction kinetic models (e.g. in combustion and atmospheric chemistry [19]). Some of these tools are applied here to the Chen's budding yeast cell cycle model in order to illustrate that the growing cell undergoes a series of irreversible transitions among cell cycle states.

## Results

### Excitation and relaxation periods during the budding yeast cell cycle

The extension of linear stability analysis to non-stationary systems tells us whether the trajectories of perturbed and unperturbed systems are converging or diverging in time (see methods). This analysis must be done all over the trajectory of the unperturbed system, because the reference point is changing in non-stationary systems [19]. If the real parts of all the eigenvalues of the linearized system are negative then the distance between the original and the perturbed systems are decreasing. If the real part of at least one of the eigenvalues is positive, then the distance between the original and the perturbed point is increasing in time. In chemical systems a positive real part eigenvalue is a sign for an autocatalytic regime when the system is in an excitation phase. In contrast when all the real part of eigenvalues are negative the system is in a relaxation period approaching to a stable steady state [19] indicating that the perturbed system approaches the unperturbed one. The imaginary part of an eigenvalue is also informative, because it determines the way (e.g. monotonous or damped oscillations) how the perturbed and the original trajectories are converging to or diverging from each other. Since we were not interested in these local characteristics of the trajectories, we have analyzed the real parts of the eigenvalues only. Therefore, in the following text term eigenvalue always refers to the real part of the complex eigenvalue.

**Figure 2:**
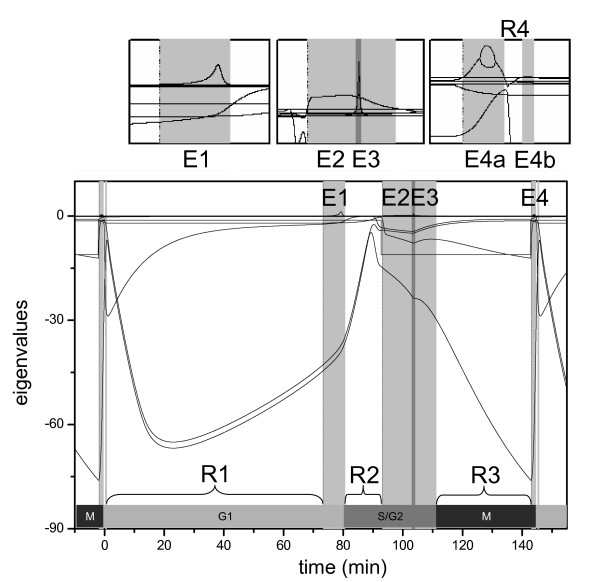
**The eigenvalues of the Jacobian during the budding yeast cell cycle**. A small daughter cell was simulated from birth (time = 0) until its subsequent cell division (time = 144.92). The grey areas mark the periods, where there is at least one positive eigenvalue. The dark grey area marks the location of a sharp positive eigenvalue peak. Cell cycle phases are noted and enlarged inlets of the positive eigenvalue regimes are attached.

The eigenvalues of the Jacobian for the Chen [12] model was calculated during the simulation of the budding yeast cell cycle (Fig. 2). There are four periods (indicated by gray shading) during the cycle (between two successive cell divisions at 0 min and at 145 mins) where at least one of the eigenvalues is positive which indicates an excitation in the underlying cell cycle control system. Excitation periods E4a and E4b are very close to each other at the end of the cycle and they are well distinguishable only on the small inlet (Fig. 2). In the middle of excitation period E2 another eigenvalue of the Jacobian becomes positive, which is indicated by dark grey shading (E3). Each excitation period is followed by a relaxation period (R1...4) where all the eigenvalues are negative.

**Figure 3:**
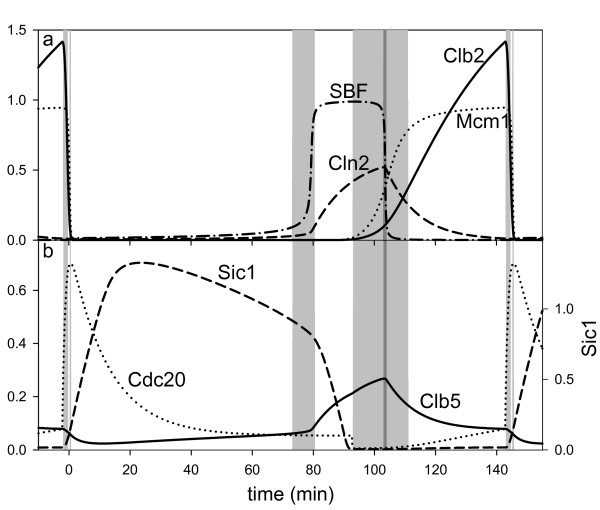
**Fluctuation in concentrations of key cell cycle regulators in the simulation of the Chen model [12]**. (a) Cln2 and SBF appear together just like Clb2 comes with Mcm1. (b) Concentrations of other key cell cycle regulators also changes at the time of positive eigenvalue periods. Background shading shows the same autocatalytic periods as on Fig. 2.

At the beginning of the cell cycle all the eigenvalues are negative and the control system is in a stable state which is called G1 phase of the cell cycle (Fig. 3). In this cell cycle phase all the major cyclins (Cln2, Clb5 and Clb2) are absent, but the level of G1 stabilizers (Sic1 and Cdh1) is high (Cdh1 is not shown). Although G1 is a stable state in the model, it is not a unique steady state, because the cell is growing in its cytoplasmic mass, which causes a slow but steady accumulation of Cdk/Cyclin activities. This is reflected in the slow decrease of Sic1 level during relaxation period R1 (Table 1) because Sic1 gets degraded due to Cdk dependent phosphorylation [17].

**Table 1:** Detailed changes in variables at excitation (E) and relaxation (R) periods of the cell cycle

	R1 G1	**E1 START**	R2 S	**E2 M entry**	**E3 growth change**	**E2 M entry**	R3 M	**E4a Ana**	R4 Telo	**E4b Exit**
***Clb2***_*T*_	—	—	—	• **/**	**/**	**/**	/	• **↓**	**↓**	**↓**
***Sic1***_*T*_	∩	**\**	\	• **—**	**—**	**—**	—	• **/**	/	**/**
***Cdh1***	—	**—**	• \	**—**	**—**	**—**	—	• **↑**	/	**—**
***Cdc20***_*T*_	\	**—**	—	**—**	**—**	• **/**	/	• **∩**	\	**\**
***Cdc20***	\	**—**	—	• **↓**	**—**	**—**	/	• **↑**	/	**∩**
***Cln2***	—	• **↑**	/	**/**	• **∩**	**\**	\	**—**	—	**—**
***Clb5***_*T*_	∪	**—**	•/	**/**	• **∩**	**\**	\	**\**	\	**\**
***SWI***	\	**—**	— • **↓**	**—**	**—**	**—**	—	• **↑**	/	**/**
***SBF***	/	• **↑**	—	— • **↓**	**↓**	**\**	—	**—**	—	• **/**
***MCM***	—	**—**	— •/	**/**	**/**	**/**	/	• **↓**	**↓**	**↓**

• major transition — no change / slow increase \ slow decrease

∩ maxima ∪ minima **↑** abrupt increase **↓** abrupt decrease

Just before 80 mins the stable G1 state looses its stability and the control system enters into excitation period E1 (Fig. 3). The positive eigenvalue of the Jacobian is the consequence of the transcriptional positive feedback loop between SBF and Cdk/Cyclin activities [14,15]. During this excitation period SBF jumps from very low value to one, which causes an increase in the rate of Cln2 and Clb5 synthesis (Table 1). Because of this positive feedback loop the cell cycle control system leaves the stable G1 state during excitation period E1 in an irreversible manner. All of these characteristics of excitation period E1 leads us to associate it with start transition of the cell cycle (Table 1).

Excitation period E1 is followed by a relaxation period (white area – R2 on Fig. 2) when the system approaches a new stable state, which is qualitatively different from G1. Since SBF is high which activates the synthesis of Cln2 and Clb5 cyclins, the level of Cln2/Cdc28 and Clb5/Cdc28 complexes is increasing. Both of these Cdk/Cyclin complexes down-regulate the G1 stabilizers (Sic1 and Cdh1) [16,18], therefore Sic1 and active Cdh1 levels are decreasing during this relaxation period (Table 1). The activity of Cln2/Cdc28 appears first which is responsible for initiation of bud formation. Since Clb5/Cdc28 is inhibited by Sic1[16], its activity appears and triggers DNA replication only after Sic1 has dropped to very low value (Fig. 3). The two cell cycle events during relaxation period R2 are initiation of bud formation and DNA replication, which are coincident during budding yeast cell cycle [3]. Therefore, we associate the state corresponding to relaxation period R2 with high SBF, Cln2, Clb5 and low Cdh1, Sic1 levels with S phase of the cell cycle (Table 1).

Relaxation period R2 is very short (12.6 mins) because the system soon enters into a new excitation period (E2). Since both Cdh1 and Sic1 are negative regulators of Cdc28/Clb2 kinase, the decrease of Sic1 and active Cdh1 levels help to activate the transcriptional positive feedback loop between Clb2/Cdc28 and Mcm1 [13]. It is true again that the transcription factor (Mcm1) for cyclin synthesis changes faster than the corresponding cyclin (Clb2) level. By entering into the second excitation period the control system shoots for states where SBF, Cln2, Clb5, Mcm1 and Clb2 levels are high, while Sic1 and active Cdh1 levels are low. Therefore we associate excitation period E2 with the irreversible decision to enter into mitosis. This excitation period interrupts relaxation period R2 (where S phase starts) which reflects the fact that budding yeast cells enter into mitosis during S phase and they do not have a real G2 phase between S and M phases [3].

There is a narrow excitation period (E3, indicated by dark grey area) in the middle of the second excitation period, which is caused by turning off the SBF-Cln2 transcriptional positive feedback loop [20]. This positive feedback was turned on during excitation period E1 at start transition. However, high Cdc28/Cln2 kinase activity – besides activating SBF (positive feedback) – also initiates a time delayed negative feedback loop: Cdc28/Cln2 down-regulates Sic1 and Cdh1, which help Cdc28/Clb2 activation which inhibits the transcription factor for Cln2 (SBF). When the rising Cdc28/Clb2 level during excitation period E2 crosses the inhibitory threshold, SBF turns off abruptly. Turning off SBF causes decrease in both Cln2 and Clb5 levels after excitation period E3 (Table 1). Since Cln2 kinase is responsible for polarized growth driving bud formation [21], this excitation changes the growth characteristic of the cell [22]. The polarized growth started at E1 by Cdc28/Cln2 switches to isotropic growth resulting in bud expansion in all directions (Table 1).

Excitation period E3 changes the characteristics of the mitotic state induced by the control system during excitation period E2. In period R3 the system relaxes to a state where Clb2 and Mcm1 levels are high, while SBF, Cln2 and Clb5 levels are low (Fig. 3 and Table 1). The excitations (E4a and E4b) drives the cells out of the mitotic state when the spindle checkpoint is released [23]. This signal stimulates Cdc20 activation that induces Clb2 degradation [24]. The drop in Clb2 level causes a decrease in Cdc28/Clb2 activity, which gets further amplified by turning off the positive feedback loop between Cdc28/Clb2 and Mcm1. Reduction of Cdc28/Clb2 kinase activity also turns the double negative feedback loop between Cdc28/Clb2 and Cdh1 to the G1 regulators side. Activation of Cdh1 causes further Clb2 degradation and an irreversible exit from mitosis. The stoichiometric Cdk inhibitor (Sic1) also gets up-regulated in an autocatalytic manner but only after some delay and it is responsible for excitation E4b. The two excitations (E4a and E4b) drive the system to a G1 state with low Clb2 level (Fig. 3 and Table 1).

### Cell cycle transitions shifted in cell cycle mutants

The cell cycle transitions described in the previous section for wild type cells are shifted in certain cell cycle mutants. The analysis of eigenvalues during simulations when the level of certain cell cycle regulators were changed helped us to understand the role of feedback loops in irreversible cell cycle transitions.

**Figure 4:**
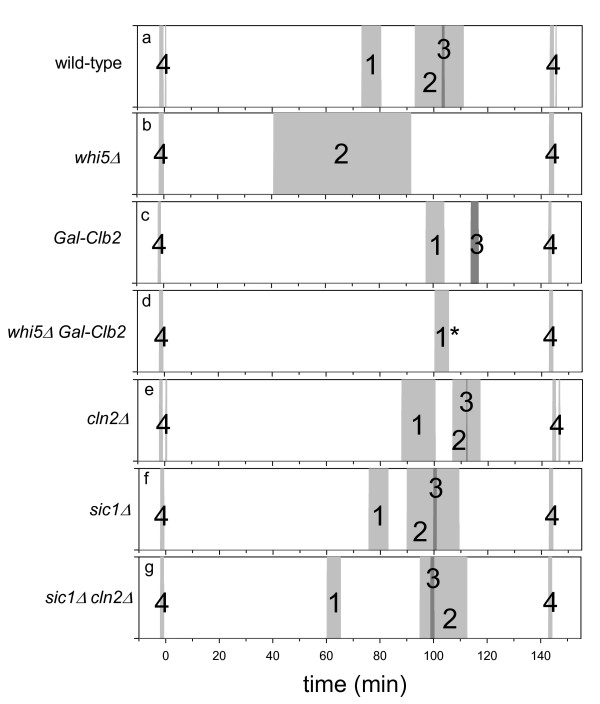
**Positive eigenvalue periods in mutant cells**. (a) wild-type (same as Fig 2); (b) *whi5*Δ (parameter changes: *k*_*isbf*_' = 0, *k*_*isbf*_" = 0); (c) *Gal-Clb2 *(*k*_*sb2*_' = 0.15); (d) *whi5*Δ *Gal-Clb2 *(see changes above); (e) *cln1*Δ *cln2*Δ (*k*_*sn2*_" = 0); (f) *sic1*Δ (*k*_*sc1*_' = 0, *k*_*sc1*_" = 0); (g) *cln1*Δ *cln2*Δ *sic1*Δ (see changes above). Excitation periods are labeled with same numbers as on Fig. 2 (1* stands for a new START related excitation period, details in text).

The SBF-Cln2 transcriptional positive feedback loop can be cancelled out by reducing SBF inhibition (*k*_*isbf*_' and *k*_*isbf*_") to zero. This parameter change corresponds to the deletion of SBF inhibitory component, Whi5, which is a viable cell [25,26]. Both E1 and E3 excitations are abolished (Fig. 4b) supporting that these excitations are the consequences of turning on and off the SBF-Cdc28/Cln2 positive feedback loop. The Cdc28/Clb2-Mcm1 transcriptional positive feedback can be eliminated by constitutive Clb2 expression which simulates a Gal-Clb2 strain, which is a viable cell [27]. Ectopic Clb2 expression eliminates excitations E2 and one of E4 (at mitotic exit) indicating that synthesis of mitotic cyclin (Clb2) is turned on and off at these excitation periods (Fig. 4c). The prediction is that in the combined double mutant, *whi5Δ Gal*-*Clb2*, four excitations should disappear, leaving one excitation period at mitotic exit intact. In contrast to our expectations, we found two excitation periods in the double mutant (see Fig. 4d). Simulations revealed that the first excitation period marks the G1/S transition where Cdh1 and Sic1 are turned off or degraded by appearing Clb2 kinase activity (not showed). This antagonism between Cdc28/Clb2 and Cdh1, Sic1 does not produce a positive eigenvalue in wild-type cells because Cdh1 and Sic1 are inactivated by Cln2 and Clb5 before Clb2 cyclin appears [18]. The situation is different in this double mutant, which cycles at a reduced cell size because of the elevated Clb2 synthesis. Although SBF is fully active, Cln2 level is small and roughly constant because the rate of synthesis is cell size dependent. As a consequence, in the double mutant it is Cdc28/Clb2 which turns off Cdh1 and eliminates Sic1 giving rise for a new excitation not observable in wild type.

In the absence of Cln2, START (E1) is delayed compared to wild type cells (Fig. 4e), because the transcriptional positive feedback loop responsible for START is compromised. This delay in START is fully compensated and START is even advanced in a *cln2Δ sic1Δ *background (Fig. 4g). Elimination of Sic1 helps Cdc28/Clb5 to activate the transcriptional positive feedback. Observe that Sic1 deletion on its own does not have an effect on the cell cycle position of START (Fig. 4f).

### Dimension of the manifold

**Figure 5:**
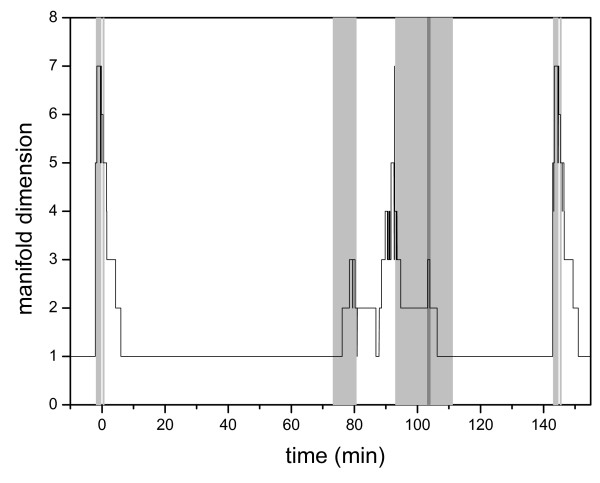
**Change of the estimated dimension of the manifold during a cell cycle**. Background shading shows the same autocatalytic periods as on Fig 2.

The dimension of the manifold of a dynamical system identifies how many variables are changing independently of the others at a certain time. Figure 5 shows the change of the dimension of the manifold during a cycle. The dimension was estimated using the theory described in methods using threshold value *z*_*thres *_= 7.0 × 10^-5^. The dimension of the manifold changes between one and seven during the cycle. The dimension usually increases in each excitation period, where one of the eigenvalues becomes positive. During excitation period E4 the dimension increases to 7 and then gradually decreases to 1; in excitation period E1 the dimension increases from 1 to 3, and there is a further a rise in dimension (up to 7) at the time of entry into mitosis, which is associated with E2 (Fig 5). The general picture is that the dimension of the manifold increases in excitation periods and then decreases in the subsequent relaxation periods. However, the dimension of the manifold never drops to zero during relaxation periods but rather to one. The minimum dimension is one because cell mass continuously increases during the cycle. Therefore, the cell cycle control system never reaches a real steady state during the cell cycle, but it is rather pulled by increasing cell mass. The fact that the model has a maximal dynamical dimension of 7 does not mean that an explicit 7-variable equivalent model could be constructed. It rather suggests that a locally valid model can be constructed with no more than 7 variables at any time point.

## Discussion

Dynamical models for natural phenomena are usually studied by computer simulations using numerical methods. The temporal patterns provided by this approach are often directly comparable to experimental data, which is a real advantage. However, computer simulations do not provide the modeler with any inside view why the mechanism works in a particular way. This disadvantage can be eliminated by using other tools of dynamical systems theory like bifurcation analysis. Bifurcation analysis provides us with recurrent solutions (steady states and oscillations) of the control system, which could be stable or unstable. The number, the nature (steady or oscillatory) and the stability of recurrent solutions are dependent on parameter values. A change in the number or the characteristic of recurrent solutions is called bifurcations. Both methods (simulations and bifurcation analysis) have been extensively used for analysis of cell cycle models. Cytoplasmic mass has an influence on the cell cycle progression, but its change is much slower than the molecular changes in the control system. Therefore cellular mass is often considered as a bifurcation parameter [7,10,28,29] which helps us to identify cell cycle states and cell cycle transitions dependent on cellular mass. However, those cell cycle transitions that are not dependent on reaching a critical cellular mass cannot be identified by bifurcation analysis.

We have tried to overcome this problem by applying a new method which has not been used for analyzing models of biological systems before. The time scale analysis applied here is a frequently applied tool for the investigation of complex chemical reaction networks (see e.g. [19]). This approach is applicable for any models which are represented by ordinary differential equations. Based on the sign of Jacobian's eigenvalues the temporal evolution of the system can be divided into excitation and relaxation periods. In addition, considering the eigenvectors of the Jacobian, the dynamical dimension of the system can also be calculated. During relaxation periods all the eigenvalues are negative and system approaches to a stable steady state. In contrast during excitation periods at least one of the eigenvalues is positive which leads to a deviation from a stable state. By applying time scale analysis to the Chen's cell cycle model [12] we have identified four excitation and four relaxation periods during the budding yeast cell cycle. The relaxation periods correspond to well known cell cycle phases like G1, S and M phases. The excitations represent the irreversible cell cycle transitions driven by activation or inactivation of positive feedback and double-negative feedback loops. The first excitation period at START transition flips the control system from a G1 state into S phase with a concomitant initiation of bud growth. The second excitation is responsible for the initiation of mitosis, which is premature in budding yeast. The third excitation changes the morphology of bud growth from polarized to isotropic. Finally the last excitation at mitotic exit pushes the system back to G1 phase. By analyzing cell cycle mutants we could determine which feedback loop is responsible for a certain excitation and cell cycle transition.

Dimension analysis revealed that the dynamical dimension of the system never exceeds seven. This finding suggests that the 13-dimensional Chen's model can be reduced to a seven dimension system at any given time point. The maximal dimension was always reached during the excitation periods and the dimension was reduced during the subsequent relaxation periods always to one. Dynamical dimension one indicates that all chemical reactions are in stationary state, and the change of the system is dictated by the increase of cell mass only.

Although limit cycle oscillators can be constructed without excitation (positive feedback) mechanisms, most examples for limit cycles are characterized by excitation-relaxation periods. In general, the dynamical dimension can be high during the whole cycle. A special category of limit cycles is based on sequential destabilization of attracting stationary points. This is the case in the Chen model, where during the relaxation periods the dimension always decreases to one. If the dimension is one, all chemical concentrations are in steady state, which is moving due to the increase of the cell mass only. Comparison of Figures 2 and 5 clearly indicates that the Chen model can be interpreted as switching between steady states.

## Conclusion

We applied mathematical tools, which have been used to investigate complex chemical reaction models before, for the analysis of a biological regulatory network. The analysis of a budding yeast cell cycle model revealed the presence of autocatalytic excitation and subsequent relaxation periods in the cell cycle and these excitation periods can be connected to major cell cycle transitions. We propose that this technique can be very useful to detect the timing and length of dynamical transitions in any mathematical model of complex regulatory biological networks.

## Methods

### Theory of the analysis

A dynamical model can be characterized by the following initial value problem

d **Y**/d *t *= **f**(**Y**,**p**),     **Y**(0) = **Y**^0 ^    (1)

where *t *is time, **Y **is the *n*-vector of variables, **p **is the *m*-vector of parameters, **Y**^0 ^is the vector of the initial values of the variables, and **f **is the right-hand-side of the differential equations. Lam and Goussis [30] and Maas and Pope [31] investigated the response of dynamical systems to the simultaneous perturbations in the values of several variables. Local characterization of a dynamical system can be based on the eigenvalue-eigenvector decomposition of the Jacobian:

**Λ **= **W J V **    (2)

where **J **= ∂**f**/∂**Y **is the Jacobian, **W **and **V **are the matrices of left and right eigenvectors, respectively; **Λ **is a diagonal matrix and its diagonal elements are the eigenvalues of the Jacobian. Denote Re(λ_*j*_) the real part of the *j*-th eigenvalue of the Jacobian, and **w**_*j *_the corresponding left eigenvector. If the values of the variables are changed by Δ*y*'^0 ^=α**w**_*j*_, where α is a small scalar, then the relaxation of the variables to the original values can be described by the following exponential function:

Δy′(t)=Δy′0eRe⁡(λj)t     (3)
 MathType@MTEF@5@5@+=feaafiart1ev1aaatCvAUfKttLearuWrP9MDH5MBPbIqV92AaeXatLxBI9gBaebbnrfifHhDYfgasaacH8akY=wiFfYdH8Gipec8Eeeu0xXdbba9frFj0=OqFfea0dXdd9vqai=hGuQ8kuc9pgc9s8qqaq=dirpe0xb9q8qiLsFr0=vr0=vr0dc8meaabaqaciaacaGaaeqabaqabeGadaaakeaacqqHuoarieqacuWF5bqEgaqbaiabcIcaOiabdsha0jabcMcaPiabg2da9iabfs5aejqb=Lha5zaafaWaaWbaaSqabeaacqaIWaamaaGccqqGLbqzdaahaaWcbeqaaiGbckfasjabcwgaLjabcIcaOiabeU7aSnaaBaaameaacqWGQbGAaeqaaSGaeiykaKIaemiDaqhaaOGaaCzcaiaaxMaadaqadaqaaiabiodaZaGaayjkaiaawMcaaaaa@460B@

According to this local linear approximation, the rate of response can be related to *n *orthogonal perturbation directions. In non-stationary systems, the reference point belonging to the unperturbed system is continuously moving in the space of variables. If Re(λ_*j*_) is negative, then the distance between the reference and the perturbed point is decreasing. The response is faster if |Re(λ_*j*_)| is larger. If Re(λ_*j*_) is zero, the trajectories of the original and perturbed systems run parallel. If Re(λ_*j*_) is positive, then the distance between the original and the perturbed point is increasing. Presence of at least a single positive eigenvalue is characteristic for a feedback regime, like the autocatalytic regime in chemical kinetic systems. For example, in models describing explosions the highest eigenvalue of the Jacobian is positive during the fast change of concentrations and all eigenvalues are negative during the subsequent relaxation period. Measures 1/|Re(λ_*j*_)| are called the timescales of dynamical systems. In nonlinear dynamical systems, the timescales depend on the values of variables and therefore on time.

Lam and Goussis [30] have investigated the presence of time scales of very different magnitude for a series of single points in the variable space. Roussel and Fraser [32] described the evolution of chemical kinetic systems in connection with slow manifolds. They stated that the existence of very different time scales in these systems causes the trajectory of the solution to move onto attracting slow manifolds. Starting from any point in the space of variables, the trajectory originally moves on a given *n' *dimensional manifold, but as time advances in the relaxation period (when all eigenvalues of the Jacobian are negative), the dynamical dimension of the movement decreases, and after some time the trajectory moves close to a two-dimensional surface (curved plate), then close to a one-dimensional curve, and finally arrives to the zero-dimensional equilibrium or stationary point if it exists. It was found [33] that in excitation periods (when at least one eigenvalue of the Jacobian is positive) the dynamical dimension of the system increases.

A simple method was suggested for the calculation of the dimension of the manifold [33,34]. In a dynamical system of *n *variables, the degree of freedom of the movement in the space of variables is *n*_1 _= *n - n*_*c*_, where *n*_*c *_is the number of conservation relations, which is equal to the number of zero eigenvalues of the Jacobian. The columns of matrix **W **indicate the basic excitation directions (called modes) in a dynamical system at a given point in the space of variables. In all these directions, distance from the stationary state *in this direction *can be calculated by equation

Δ*z_j _*= **w**_*j*_**f**/Re(λ_*j*_)     (4)

If the system is close to the stationary state with respect to mode *j*, that is if Δ*z*_*j *_is smaller than threshold *z*_*thres*_, the system is in the stationary point of the corresponding direction. Let *n*_*r *_denote the number of such so called relaxed modes. The actual dynamical dimension of the system is *n*_*D *_= *n *- *n*_*c *_- *n*_*r*_. For a justification and details see [34].

### Simulation methods

The cell cycle model was simulated using the equations, parameters and initial values given by Chen *et al*. [12]. Due to the zero initial values for several enzyme concentrations, the beginning of the first period was different from the others, but the concentration changes during all subsequent periods were identical. In all simulations the daughter cell was followed [12]. Following the original model, we used variable *Cln2 *representing the sum of Cdc28/Cln1 and Cdc28/Cln2 complexes, *Clb5 *for Cdc28/Clb5 and Cdc28/Clb6, and *Clb2 *for Cdc28/Clb1 and Cdc28/Clb2.

In all figures in this paper, the time dependent values were plotted for a whole cell cycle (144.92 minutes) plus ten minutes before and after that period. Time zero marked the division of the cell. The results presented here were calculated by specific goal oriented Fortran codes derived from the KINAL and KINALC packages [35], which is downloadable from our website [36].

## Abbreviations

Cdk – Cyclin dependent kinase; SBF – SCB binding factor; MBF – MCB binding factor

## Authors' contributions

IGZ and JZ did the early calculations, while AL carried out the final computations. TT suggested the application of time scale and dimension analysis for the investigation of the cell cycle model. ACN and BN analyzed the correlation between the mathematical results and the published experimental observations and initiated the analysis of mutants. ACN, TT and BN drafted the manuscript. All authors read and approved the final manuscript.
